# Immunization Dose-Dependent In Vivo Replication Kinetics and Protective Efficacy of the Gene-Deleted Marek’s Disease Vaccine SC9-1

**DOI:** 10.3390/v18101052

**Published:** 2026-09-22

**Authors:** Rujun Ye, Ruihan Shi, Haijun Jiang, Chang Liu, Mengzhu Dong, Hairong Tian, Tingwen Zheng, Yu Zhou

**Affiliations:** Institute of Animal Husbandry and Veterinary Medicine, Beijing Academy of Agriculture and Forestry Sciences, Beijing 100089, China

**Keywords:** Marek’s disease, gene-deleted vaccine, SC9-1, immunization dose, in vivo replication kinetics, protective efficacy, SPF chicken

## Abstract

In this study, we evaluated the replication kinetics and protective efficacy of the gene-deleted Marek’s disease (MD) vaccine strain SC9-1 in specific-pathogen-free (SPF) chickens immunized with different doses. Chickens were divided into two groups: one group received a high dose (5000 PFU) and the other a low dose (500 PFU) of the vaccine at 1 day of age, with a non-vaccinated group serving as the control. Vaccine viral copy numbers in the spleen and feather tips were detected by quantitative PCR (qPCR) at different days post-vaccination (dpv) (3, 5, 7, 11, and 14 dpv). Meanwhile, a challenge trial was conducted where chickens were challenged with the very virulent Md5 strain (1000 PFU/bird) at 5 dpv and observed until 70 days of age. Results from the vaccination-only experiment revealed that viral replication in the spleen was detectable in 100% of chickens as early as 3 dpv, reaching its peak at 5 dpv, with the high-dose group exhibiting significantly higher viral loads than the low-dose group. In feather tips, replication was detectable in 100% of chickens by 5 dpv and peaked at 7 dpv, but no significant difference was observed between the two dose groups. The unvaccinated control group exhibited 100% mortality with characteristic gross and histopathological lesions of Marek’s disease. In stark contrast, both vaccinated groups (high and low dose) demonstrated 100% survival until the end of the observation period (70 days). No clinical signs or pathological abnormalities were observed in these groups. Furthermore, vaccination with SC9-1 did not adversely impact growth performance and the development of major immune organs, even following subsequent Md5 challenge. The results indicated that a higher initial vaccination dose of the SC9-1 vaccine was associated with elevated peak early in vivo replication level, whereas vaccination dose may not govern viral maturation and shedding levels of SC9-1 in feather-tip tissues. However, peak replication level was not a direct determinant of final protective efficacy. Notably, even the lowest tested dose (500 PFU) afforded full protection against early challenge with a very virulent MDV strain. Collectively, these observations contribute to our understanding of dose-dependent protection induced by gene-deleted MD vaccines and inform rational vaccination strategies.

## 1. Introduction

Marek’s disease, caused by the highly contagious oncogenic herpesvirus Gallid alphaherpesvirus 2, is a lymphoproliferative disease of chickens that causes substantial economic losses for the global poultry industry [1,2,3,4]. Vaccination constitutes the primary cornerstone for MD control. Since the 1970s, live vaccines including the turkey herpesvirus (HVT) FC-126 strain (serotype 3) and CVI988/Rispens (serotype 1) have been widely deployed in poultry production [5]. Nevertheless, ongoing virulence evolution of circulating MDV has given rise to very virulent (vv) and very virulent plus (vv+) strains, which pose substantial challenges to the protective performance of existing commercial vaccines [6,7,8,9,10]. The SC9-1 strain is a novel gene-deleted live vaccine derived from the vvMDV field isolate GX0101 via deletion of the meq oncogene using bacterial artificial chromosome (BAC) and homologous recombination technology [11]. This meq-gene deletion attenuates viral pathogenicity while preserving immunogenicity. Previous experimental evaluations have demonstrated that SC9-1 exhibits a favorable safety profile without impairing the growth of vaccinated chickens, and confers superior protection against very virulent Md5 challenge compared with the CVI988/Rispens vaccine [12].

For live-attenuated Marek’s disease vaccines, in vivo viral replication is generally regarded as a fundamental prerequisite for the establishment of protective immunity [13]. Studies on conventional MD vaccines, including CVI988/Rispens and various HVT-based vaccine platforms, have revealed distinct dose–response patterns, and pronounced inter-animal heterogeneity in vaccine replication is frequently observed under sub-optimal vaccination-dose conditions [14,15]. While abundant experimental evidence has been generated from classical attenuated serotype-1 and HVT-vector vaccines to advance our understanding of dose-dependent immune responses, comparable systematic investigations exploring the inter-relationship among immunization dose, in vivo replication kinetics, and protective efficacy remain largely scarce for gene-deleted recombinant MD vaccine candidates [14,15,16,17].

Although the protective capacity of SC9-1 has been documented, how vaccination dose shapes its early in vivo replication dynamics and subsequent protective immunity has not been thoroughly characterized. This study aimed to characterize replication kinetics of the gene-deleted SC9-1 vaccine at two immunization doses (500 PFU and 5000 PFU) in SPF chickens and assess its protective efficacy against early challenge with the vvMDV Md5 strain. These findings will provide experimental support for rational clinical application of SC9-1 and add new insights into the immune mechanisms of gene-deleted MD vaccines.

## 2. Materials and Methods

### 2.1. Virus and Experimental Animals

The gene-deleted live vaccine strain SC9-1 manufactured by Beijing Lingyu Biotechnology Co., Ltd., Beijing, China was used in the present study. All vaccination procedures were performed in strict accordance with the manufacturer’s specifications and the relevant requirements issued by the Ministry of Agriculture and Rural Affairs of China. The very virulent MDV strain Md5 was kindly provided by Professor Zhizhong Cui (College of Animal Science, Shandong Agricultural University, China). One-day-old SPF chickens were purchased from Beijing Boehringer Ingelheim Viton Biotechnology Co., Ltd., Beijing, China.

### 2.2. Ethics Statement

All animal experiments were approved by the Animal Ethics Committee of the Institute of Animal Husbandry and Veterinary Medicine, Beijing Academy of Agriculture and Forestry Sciences, China.

### 2.3. Real-Time Quantitative PCR Detection and Standard Curve Establishment

Based on the genomic sequence of the SC9-1 parental wild-type strain GX0101 (GenBank accession no.: JX844666.1), specific primers targeting the viral gB gene were designed for SYBR Green-based real-time quantitative PCR analysis (Table 1). The full-length gB gene was amplified from SC9-1 genomic DNA, cloned into the pCE2-T vector, and transformed into DH5α competent cells. Positive recombinant plasmids were extracted and quantified using a microspectrophotometer. The plasmid copy number was calculated and serially diluted with ddH_2_O to generate standard gradient solutions ranging from 10^1^ to 10^9^ copies/μL.

A standard curve was established by plotting the logarithmic value of the initial plasmid copy number (lgC) against the cycle threshold (Ct) values. The correlation coefficient (R^2^) and amplification efficiency (E) were calculated to verify the accuracy and reliability of the established qPCR quantitative system.

### 2.4. Animal Immunization and Viral Replication Kinetics

A total of 45 one-day-old SPF chickens were randomly allocated into three groups (*n* = 15 per group). Chickens in the high-dose group (Q1) received subcutaneous neck immunization with 5000 PFU of the SC9-1 vaccine, while those in the low-dose group (Q2) were immunized with 500 PFU of SC9-1 at 1 day of age. The untreated group (N) received no sham injection and served as the non-immunized blank control. Notably, 5000 PFU represents the commercially recommended vaccination dose for SC9-1 under practical poultry production conditions. The 500 PFU dose corresponds to a 10-fold dilution of the standard dose, which was used to evaluate whether SC9-1 could retain protective capacity under reduced-dose conditions.

All chickens were housed in separate sterile isolators, with one isolator assigned exclusively to each experimental group. SPF chicks used in this study were straight-run (mixed-sex). Birds received commercial SPF-chick-specific feed with age-adjusted feeding rations: from 1 to 9 days of age, the daily feed supply (in grams) equaled bird age plus 2 g; from 10 to 19 days of age, bird age plus 1 g; from 20 to 49 days of age, the daily ration matched bird age; from 49 to 70 days of age, daily feed supply was calculated as 50 g plus half the number of days exceeding 50 days of age. Drinking water was provided ad libitum. A full-spectrum lighting regime was used: continuous illumination was maintained for young chicks, and the photoperiod was gradually shortened as the birds aged. At 3, 5, 7, 11, and 14 days post-vaccination, three chickens per group were randomly selected and humanely euthanized via carbon dioxide inhalation for tissue collection. No chicken was repeatedly sampled across multiple time points; distinct animals were used at each sampling time point. Spleen and feather-tip tissues were harvested for total DNA extraction. Feather-tip samples were collected from wing feathers: two feathers were collected from the left wing and two from the right wing of each chicken, retaining approximately 0.5 cm of tissue at the feather-calamus base. For nucleic acid extraction, approximately 30 mg of spleen tissue and four wing-feather tips per chicken were processed. A total of 300 μL of PBS was added to each spleen sample and 500 μL PBS to each feather-tip sample. Tissues were homogenized using a tissue homogenizer operated at 60 Hz for 30 s over 3–5 cycles. A 300 μL aliquot of the tissue homogenate was used for genomic DNA isolation employing the Virus DNA Extraction kit (Vazyme Biotech Co., Ltd., Nanjing, China), performed strictly according to the manufacturer’s instructions. Viral genome copy numbers in spleen and feather-tip tissues were quantified by qPCR to characterize in vivo replication kinetics of the vaccine strain.

### 2.5. Evaluation of Vaccine Protective Efficacy Against Md5 Challenge

A separate virulent challenge experiment was performed to evaluate the protective efficacy of SC9-1. Forty one-day-old SPF chickens were randomly divided into four groups (*n* = 10 per group): S1 (high-dose immunization + Md5 challenge), S2 (low-dose immunization + Md5 challenge), M (non-immunized Md5 challenge control), and N (blank control). Housing and husbandry conditions for animals in this challenge trial were identical to those described in Section 2.4.

Chickens in groups S1 and S2 were subcutaneously immunized with 5000 PFU and 500 PFU of SC9-1 at 1 day of age, respectively. All chickens in groups S1, S2, and M received an intraabdominal challenge with 1000 PFU of the very virulent Md5 strain at 6 days of age (5 dpv), while group N received no sham injection and remained untreated throughout the experiment.

All experimental chickens were monitored daily for clinical symptoms and mortality (defined as cumulative animal removal by humane-endpoint euthanasia; no spontaneous death from infection occurred in this study) until 70 days of age. Animals exhibiting severe clinical signs, including marked depression, paralysis, severe weight loss, or inability to access feed and water, were humanely euthanized via carbon dioxide inhalation and necropsied to examine typical macroscopic MD lesions [1]. All surviving chickens were humanely euthanized at 70 days of age and subjected to gross pathological examination. The body weight of each chicken was recorded, and the spleen and bursa of Fabricius were dissected and weighed to calculate corresponding immune organ indices, calculated as organ weight divided by body weight and expressed as percentage of body weight (%BW). Spleen specimens for histopathological evaluation were collected from one animal of group M that was humanely euthanized due to severe clinical signs at 60 days of age, and from asymptomatic animals of groups N, S1 and S2 humanely euthanized at the scheduled experimental endpoint of 70 days of age. The spleen was selected as the primary target organ for microscopic pathological evaluation owing to its central role in MDV replication and lesion development. Microscopic examination of peripheral nerves and other visceral organs was not established as an experimental endpoint in the present study. All spleen specimens were fixed in 10% neutral-buffered formalin, processed for paraffin embedding, sectioned, and stained with hematoxylin–eosin (H&E) for histopathological evaluation.

### 2.6. Statistical Analysis

All statistical analyses were performed using GraphPad Prism 8.0 software (GraphPad Software, San Diego, CA, USA). Given that chickens were humanely euthanized for tissue collection and independent animal subsets were sampled at each post-vaccination time point, one-way analysis of variance (ANOVA) was used for intergroup comparisons of viral loads, body weight, and immune organ indices at individual time points. A *p* value < 0.05 was considered statistically significant, while *p* value < 0.01 was defined as highly significant.

## 3. Results

### 3.1. Real-Time qPCR Standard Curve

Serial dilutions of plasmid standard were used to construct the standard curve, giving a linear regression equation of Y = −3.352X + 37.710 (Figure 1). The assay achieved 98.8% amplification efficiency together with a correlation coefficient of 0.996. The near-optimal amplification efficiency and excellent linear correlation verify satisfactory analytical performance, supporting high-sensitivity, dependable absolute quantification of SC9-1 viral loads using this standard curve.

### 3.2. Replication Kinetics of SC9-1 in Spleen

One-day-old chickens received subcutaneous neck immunization with 5000 PFU (group Q1, high-dose) or 500 PFU (group Q2, low-dose) of the SC9-1 vaccine. qPCR-monitored SC9-1 replication in the spleen exhibited similar kinetic profiles between the two dose groups (Table 2, Figure 2A,B). The SC9-1 virus was detectable in all three sampled chickens from both Q1 and Q2 as early as 3 days post-vaccination. Although higher vaccine genome loads were observed in the high-dose Q1 group compared with the low-dose Q2 group at this time point, the difference was not statistically significant. Viral replication peaked at 5 days post-vaccination for both groups. At this peak time point, splenic viral loads were significantly higher in the high-dose (5000 PFU) group than in the low-dose (500 PFU) group (** *p* < 0.01). The mean splenic vaccine viral load in the high-dose group reached 10^6^ copies, approximately four-fold higher than the corresponding mean value in the low-dose group (Table 2). Following the peak, viral loads gradually declined in both groups, and no inter-group significant differences were observed at the remaining sampling time points (7, 11 and 14 dpv). No SC9-1 viral genomes were detected in spleen and feather-tip tissues from non-immunized blank control group N.

### 3.3. Replication Kinetics of SC9-1 in Feather Tips

In feather-tip tissues, SC9-1 virus was undetectable in both groups at 3 days post-vaccination (Table 2, Figure 2C,D). The virus was first detected at 5 days post-vaccination and reached peak levels at 7 days post-vaccination, before viral loads had declined by 14 days post-vaccination. In contrast to splenic observations, no statistically significant inter-group differences in feather-tip viral loads were identified between the high-dose and low-dose groups across all sampled time points. Notably, consistently low viral loads were maintained in feather-tip tissues throughout the entire monitoring period.

### 3.4. Evaluation of Protective Efficacy Against Challenge with Md5 Strain

The protective efficacy of the vaccine was assessed by monitoring survival rates and gross pathological lesions following challenge with the very virulent MDV strain Md5. As illustrated in Figure 3A, chickens in the challenge control group (Group M) began to develop severe clinical Marek’s disease-associated signs from 24 days of age, reaching 100% mortality by 60 days of age. In stark contrast, chickens in both the high-dose (S1, 5000 PFU) and low-dose (S2, 500 PFU) immunized groups exhibited 100% survival throughout the 70-day observation period, demonstrating complete protection conferred by vaccination. Gross necropsy observations further corroborated these survival data (Figure 3B–E). Euthanized chickens in Group M developed characteristic Marek’s disease-associated gross lesions, mainly marked by prominent visceral organ enlargement and multiple diffuse tumor nodules (arrows, Figure 3B). By contrast, none of the chickens from the negative control group (Group N) or the two vaccinated groups (S1 and S2) developed MD-typical clinical signs or gross lesions throughout the study (Figure 3C–E). The lack of macroscopic changes in these groups indicated that vaccination effectively prevented overt MD.

Microscopic examination of splenic tissues was performed to evaluate vaccine-mediated protection against virulent MDV challenge. As shown in Figure 4A, spleens from challenge control (Group M) chickens displayed severe architectural disruption, including marked edema, extensive parenchymal necrosis, abundant fibrin exudation, and loss of normal tissue organization. Diffuse lymphocyte infiltration and hyperplasia were evident, alongside atypical cellular morphology and visible pathological mitotic figures (arrow), consistent with classic histopathological features of Marek’s disease. In comparison, the negative control group (Group N) and both vaccinated groups (S1: high-dose, 5000 PFU; S2: low-dose, 500 PFU) retained well-preserved splenic microarchitecture (Figure 4B–D). Clear demarcation between red pulp and white pulp was maintained, with parenchymal cells densely and orderly arranged. Notably, splenic sections from the two vaccinated and challenged groups exhibited comparatively denser lymphocyte populations relative to the negative control group, which reflected normal immune activation after vaccination. No significant pathological abnormalities were detected, indicating an absence of MDV-mediated tissue damage.

Evaluation of growth and immune organ development at 70 days of age revealed no significant differences in average body weight, spleen index, or bursa of Fabricius index among groups S1, S2 and N (Figure 5). These findings indicate that Md5 challenge exerted no adverse effects on growth performance and the development of major immune organs in chickens immunized with either high- or low-doses of the SC9-1 vaccine.

## 4. Discussion

This study systematically characterized the in vivo replication dynamics of the SC9-1 vaccine strain in SPF chickens. Higher vaccination doses resulted in elevated peak viral replication levels in the spleen. Specifically, at 5 dpv, the high-dose group (5000 PFU) exhibited significantly higher splenic viral loads than the low-dose group (500 PFU). As a primary site for early MDV-mediated cytolytic infection and immune initiation, the spleen determines the magnitude and kinetics of early host antiviral responses. A higher vaccine inoculum delivers more viral particles to splenic target cells, thereby promoting robust innate immune activation and priming subsequent adaptive immune responses. Notably, vaccination dose did not alter the temporal pattern of viral replication; the peak splenic viral load consistently occurred at 5 dpv in both dose groups. Furthermore, SC9-1 displayed rapid and efficient host colonization, achieving 100% viral detection in the spleen at 3 dpv and in feather-tip tissues at 5 dpv. This early tissue tropism is superior to that of the conventional CVI988 vaccine strain, as reported in previous studies [6,18,19,20], indicating that SC9-1 possesses prominent early replication advantages that are critical for protecting young chicks from early exposure to vv-MDV.

Despite the dose-dependent enhancement of early splenic replication, both high- and low-dose SC9-1 immunization provided complete protection against vv-Md5 challenge, as consistently validated by survival rates and pathological examination. Microscopic observation of splenic tissue sections revealed denser lymphocyte distribution in vaccinated and challenged chickens compared with non-vaccinated controls, indicating effective immune activation induced by SC9-1 vaccination. This enhanced lymphocytic response contributes to the establishment of solid protective immunity against vv-MDV challenge. In addition to survival and pathological evaluation, growth performance and immune organ indices served as auxiliary indicators to comprehensively assess vaccine protection. Consistent with previous reports showing that insufficient MD vaccine protection typically leads to significant growth retardation after virulent challenge [14], the present study demonstrated that body weight, spleen index, and bursa of Fabricius index were well maintained in both vaccinated groups at 70 dpv. The intact growth status and normal development of immune organs further confirm that both 500 PFU and 5000 PFU SC9-1 immunization confer comprehensive protection against vv-MDV infection.

A distinct dose plateau protective phenomenon was observed in SC9-1, which is consistent with the immunological characteristics of classical serotype 1 MD vaccines such as CVI988. Previous studies have verified that a reduced dose of CVI988 can generate equivalent or even superior protection compared with the full standard dose [13], strongly supporting the existence of a minimum protective dose threshold for serotype 1 MD vaccines. Excessively high vaccination doses do not produce additional protective benefits and may unnecessarily increase host immune burden and field production costs. Of note, our present SPF-chicken-based study mainly focused on clinical manifestations, viral replication dynamics and protective efficacy against virulent MDV challenge, without systematic assessment of early-phase lymphoid organ pathological alterations. The 5000 PFU high dose used in this work corresponds to the recommended field dose of the SC9-1 vaccine, which has been widely deployed in multiple provinces across China. To date, field adverse events suggestive of such early lymphoid organ injuries have not been documented under practical poultry production conditions. Notably, systematic early-phase lymphoid organ pathological assessments under elevated-dose vaccination regimens are also absent for the classical serotype 1 MD vaccine CVI988. Nevertheless, further dedicated laboratory studies are still warranted to comprehensively characterize the early-stage lymphoid-associated safety profile of such vaccines under controlled experimental settings.

The present splenic replication data further verify that the magnitude of in vivo viral replication alone cannot serve as a sole predictive biomarker for vaccine protective efficacy. Findings from serotype 1 MD-vaccine experimental studies by Gimeno underscore that viral replication levels alone are insufficient to define vaccine protective potency, with multiple host–virus interactions jointly contributing to the overall protective outcome [16]. In addition to viral replication kinetics, early host immune activation, particularly T-cell-dependent responses, plays an indispensable role in vaccine-mediated protection.

Interestingly, the dose-dependent replication enhancement observed in the spleen was not detected in feather-tip tissues, indicating tissue-specific replication characteristics of SC9-1. The dose-independent replication pattern in feather tips provides a reasonable mechanistic explanation for the stable low-dose protection of SC9-1. The feather follicle epithelium (FFE) is the definitive tissue for MDV terminal maturation and horizontal transmission. Viral accumulation in the FFE is primarily governed by systemic viral trafficking efficiency and local in situ replication rather than initial inoculation dosage. Accordingly, once SC9-1 successfully colonizes primary lymphoid organs and establishes systemic dissemination, vaccine-induced protective immunity plateaus and cannot be further improved by dose escalation.

Distinct from conventional CVI988, SC9-1 maintains consistently low feather-tip viral replication throughout the entire experimental period, a unique phenotype observed upon *meq*-gene deletion in this recombinant backbone. The restricted replication in feather tissues represents a key phenotypic difference between SC9-1 and its parental wild-type strain GX0101. However, this replication-limiting trait is not universally observed in other *meq*-knockout MDV strains [21], suggesting that this phenotype is strain-specific and may be modulated by additional genetic elements inherent to SC9-1. Importantly, the low feather-tip replication profile of SC9-1 markedly reduces the potential for vaccine virus shedding, thereby conferring superior biosafety advantages for field application.

MD vaccine serotypes exhibit dramatic differences in dose dependency. Serotype 3 HVT vaccines are highly dose-sensitive, and any reduction in immunization dosage will significantly compromise protective efficacy [15]. In contrast, serotype 1 MD vaccines (including CVI988 and SC9-1) exhibit stronger tolerance to dose variation. The excellent low-dose protection of SC9-1 is closely related to its genetic background. Derived from the wild-type field strain GX0101—a naturally circulating MDV strain characterized by robust horizontal transmission capacity [22,23]—the *meq*-deleted SC9-1 may retain the parental strain’s superior early in vivo replication ability and intrinsic immunogenicity, enabling effective immune priming even at a low inoculation dose.

Collectively, the present study demonstrates that higher vaccination dosage promotes elevated early splenic replication of SC9-1, while revealing a prominent non-linear dose–protection relationship. Within the tested range of 500–5000 PFU, SC9-1 induces rapid and robust splenic replication and achieves complete protection against vv-Md5 challenge, with protective efficacy reaching a stable plateau at 500 PFU. These findings provide novel mechanistic insights into the immune characteristics of gene-deleted MD vaccines, enrich the theoretical basis for MD vaccine dose effect mechanisms, and offer precise guidance for the optimized clinical application of SC9-1.

This study has certain limitations. First, the missing feather-tip samples at 11 dpv limit the complete profiling of viral replication kinetics, making it impossible to fully exclude potential inter-group differences or a shifted replication peak at this time point. Second, all experiments were conducted in SPF chickens free of MDV-specific maternal antibodies. Considering the prevalent interference of maternal antibodies in commercial poultry farms, further validation in field-relevant chicken populations is required to verify the practical applicability of the minimum effective dose defined in this study.

## Figures and Tables

**Figure 1:**
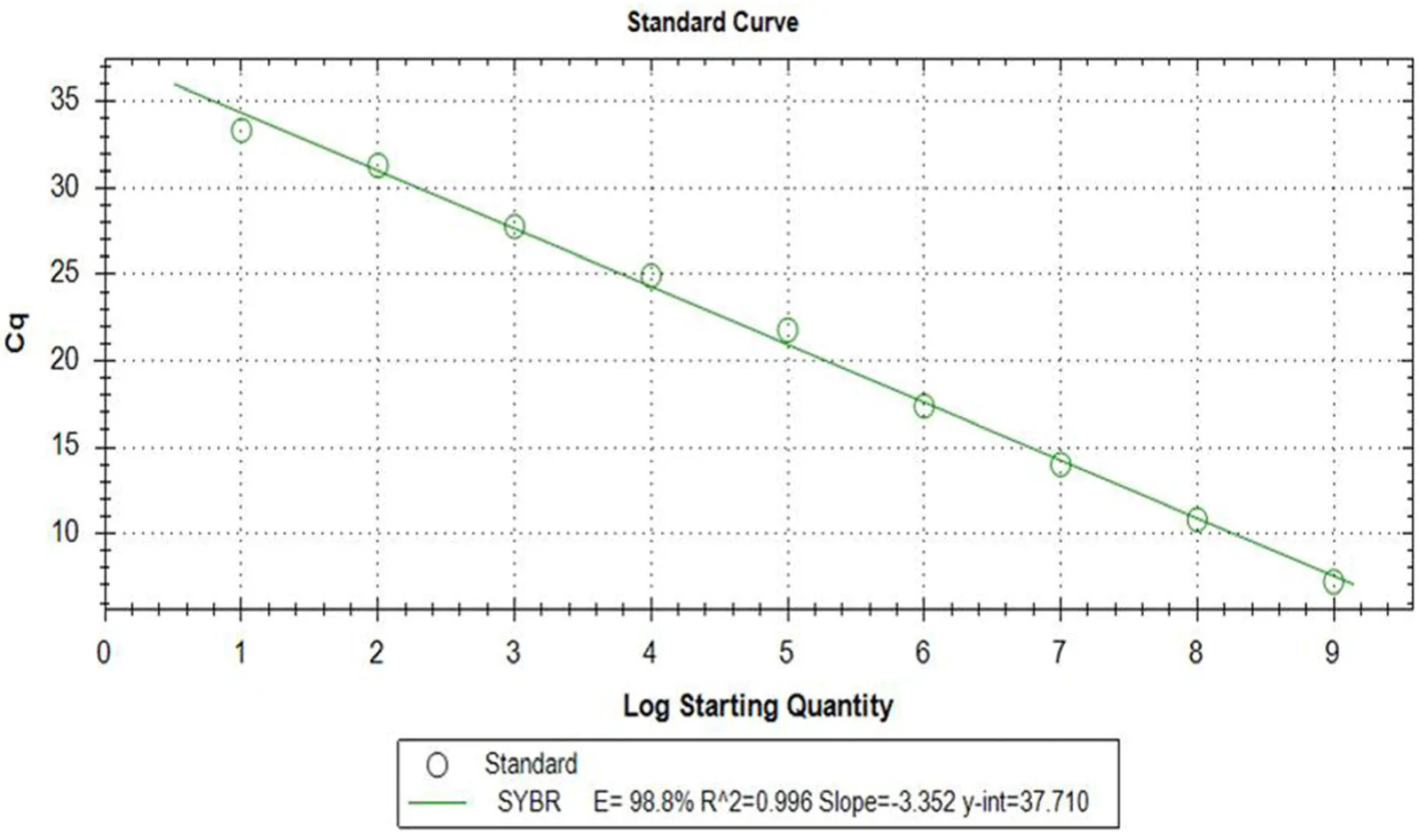
Standard curve of real-time qPCR. The standard curve generated from the serial dilutions of the plasmid (ranging from 10^1^ to 10^9^ copies/μL) showed a linear regression equation of Y = −3.352X + 37.710, with a correlation coefficient of 0.996. The amplification efficiency was 98.8%. Quantification of the SC9-1 viral load in tissue samples was achieved based on the established standard curve.

**Figure 2:**
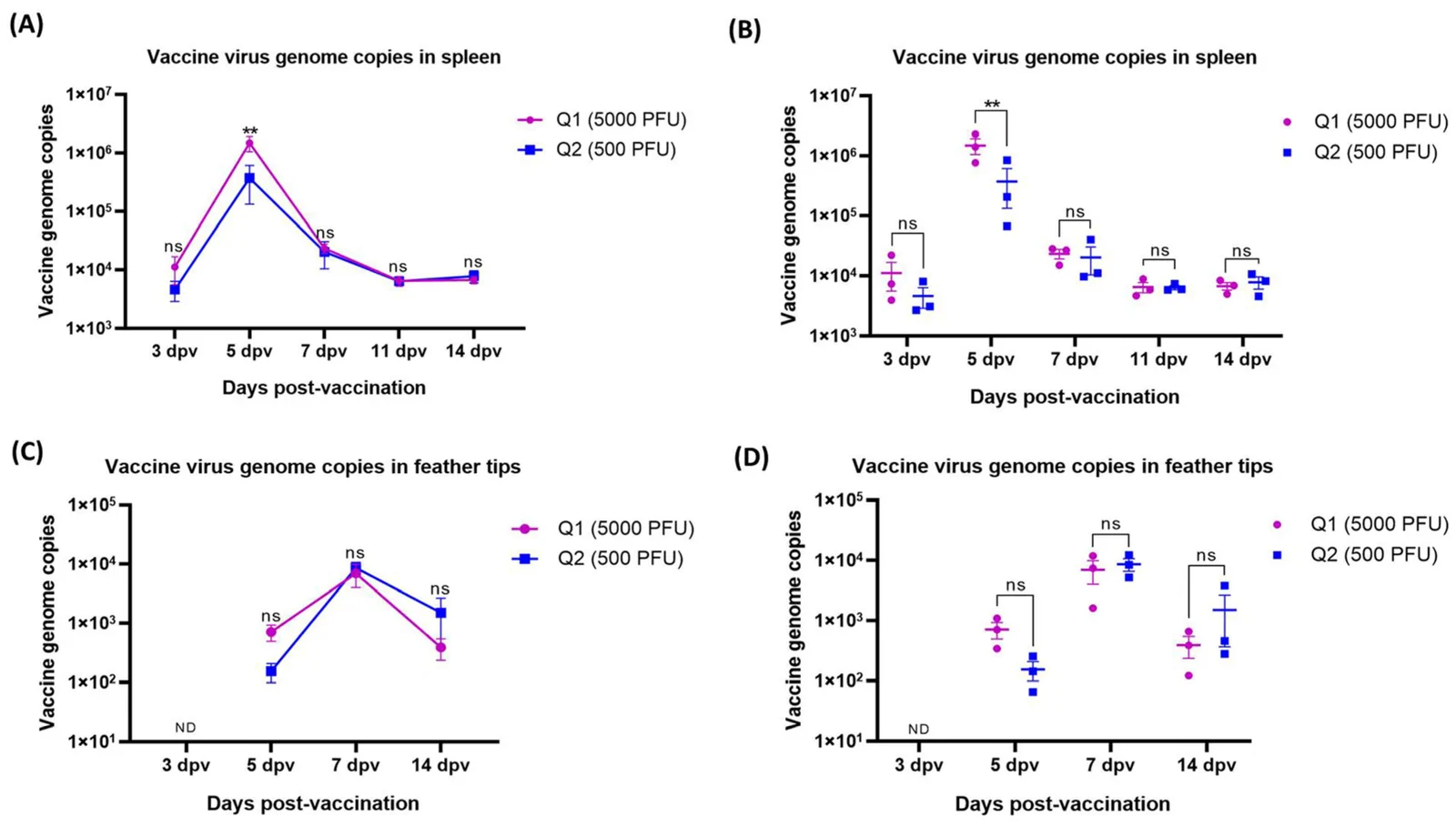
In vivo replication kinetics of SC9-1 vaccine in spleen and feather-tip tissues of SPF chickens. One-day-old SPF chickens were subcutaneously immunized with 5000 PFU (Q1, high-dose group) or 500 PFU (Q2, low-dose group) SC9-1 vaccine. At 3, 5, 7, 11, and 14 dpv, three chickens per group were humanely euthanized for tissue collection. SC9-1 viral genome copy numbers (copies per 10^5^ host cells) in spleen and feather-tip samples were quantified by qPCR. Panels (**A**,**C**) are line graphs presenting mean viral loads with standard error of the mean (SEM); panels (**B**,**D**) are scatter plots displaying individual animal data overlaid with mean ± SEM. (**A**,**B**) Vaccine viral loads in the spleen. (**C**,**D**) Vaccine viral loads in feather-tip tissues. The non-immunized blank control group (N) was not included, as SC9-1 viral genomes were consistently undetectable in this group. Feather-tip tissue samples at 11 dpv were not available. ND, not detected; **, *p* < 0.01; ns, not significant.

**Figure 3:**
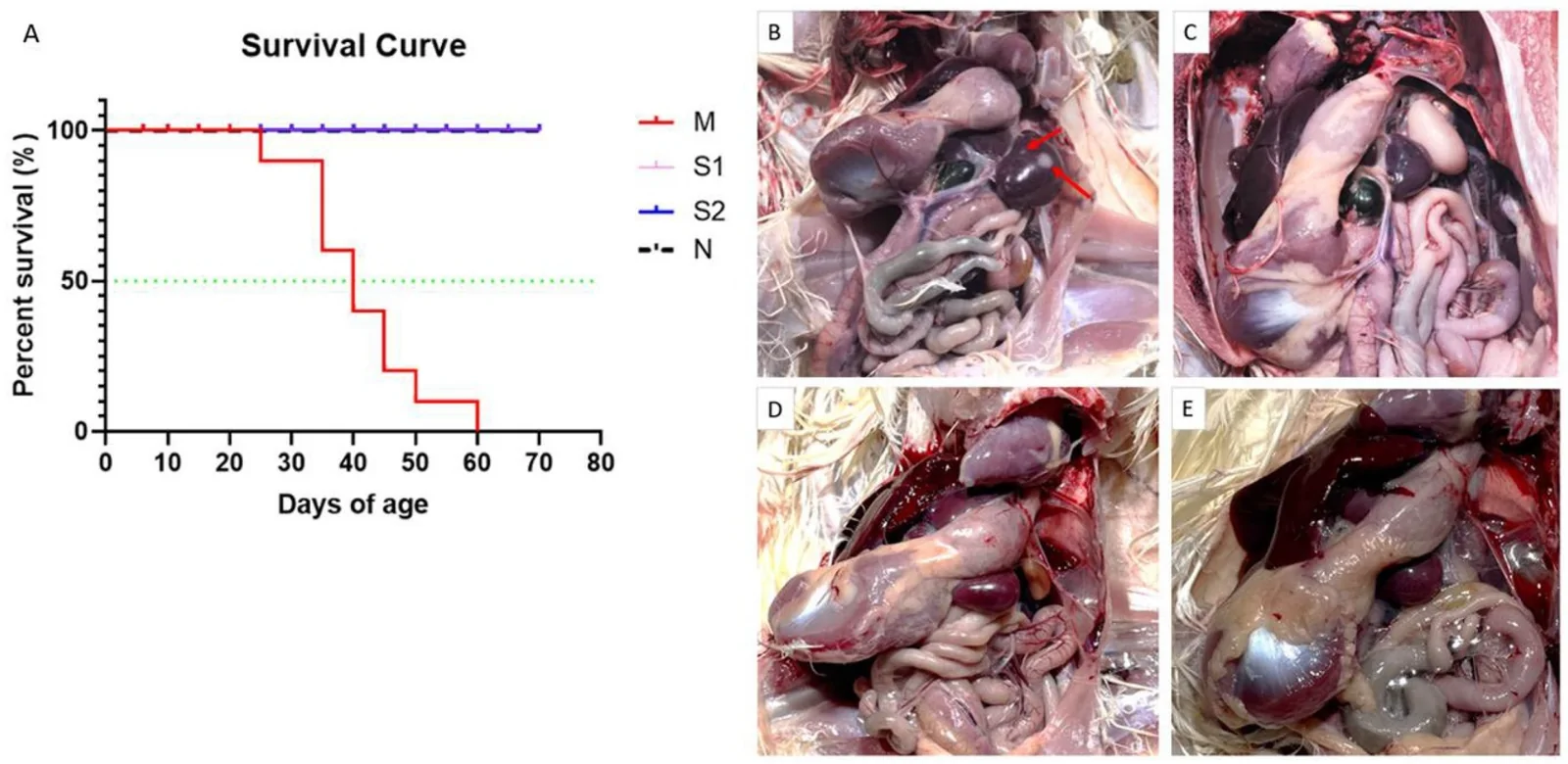
Evaluation of protective efficacy in chickens following the immunization-challenge trial. Representative gross necropsy photographs are shown. Necropsy images in panel (**B**) were obtained from a group M animal humanely euthanized due to severe clinical signs at 60 days of age; panels (**C**–**E**) show gross findings from animals humanely euthanized at the scheduled experimental endpoint of 70 days of age, representing groups N, S1 and S2. (**A**) Survival curve. (**B**–**E**) Gross necropsy observations of representative chickens from each group. (**B**) Group M (challenge control): Typical Marek’s disease lesions were observed, characterized by marked visceral organ enlargement and multiple tumor nodules (arrows). (**C**) Group N (negative control). (**D**) Group S1 (high-dose vaccine, 5000 PFU). (**E**) Group S2 (low-dose vaccine, 500 PFU). No chickens in groups N, S1, or S2 exhibited clinical signs or gross lesions characteristic of MD.

**Figure 4:**
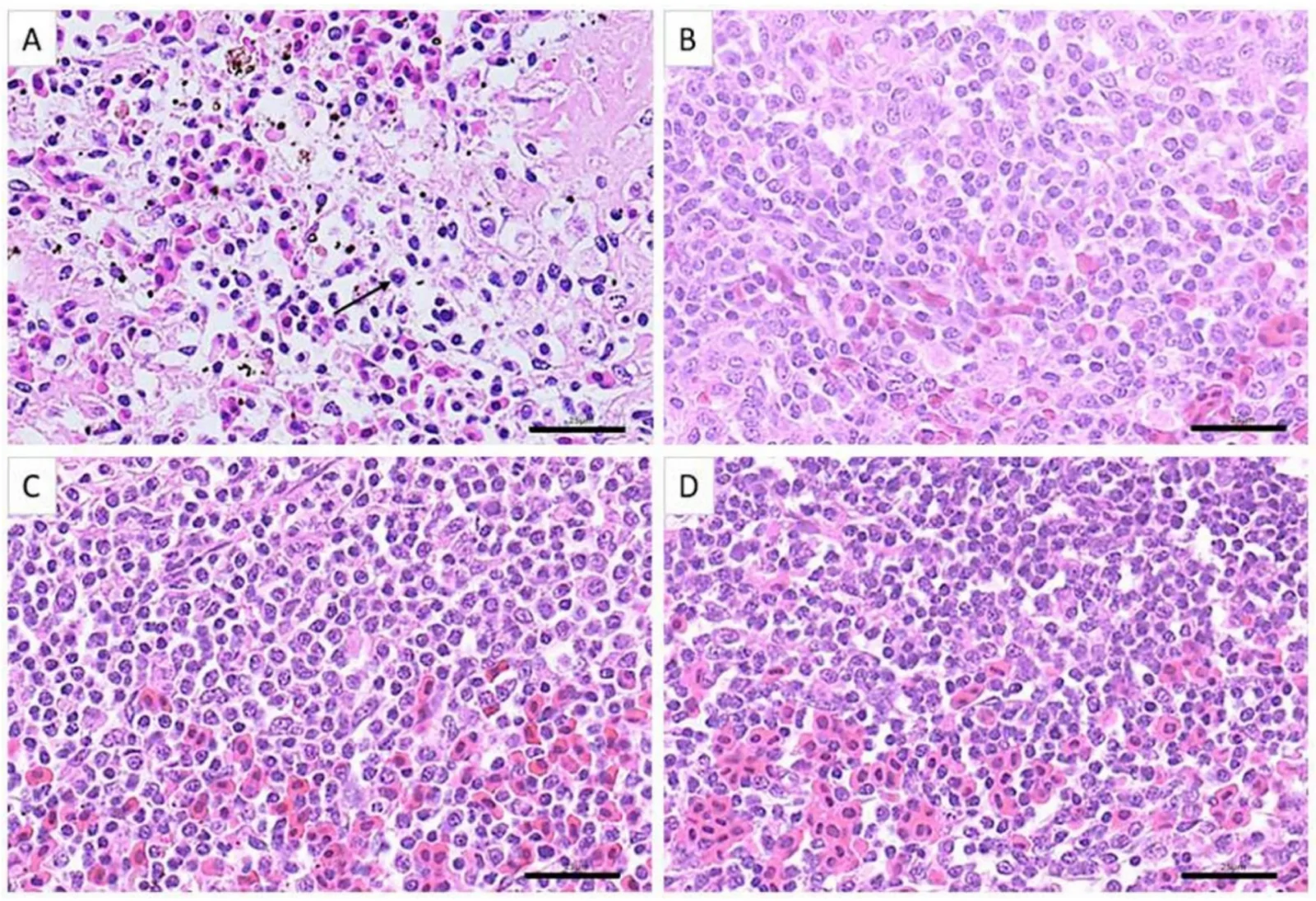
Microscopic analysis of splenic tissues from chickens in the immunization-challenge protection trial (H&E staining). Representative splenic micrographs are shown. Spleen specimens for microscopic examination were collected from a group M animal humanely euthanized due to severe clinical signs at 60 days of age, and from animals humanely euthanized at the scheduled experimental endpoint of 70 days of age from groups N, S1 and S2. (**A**) Group M (challenge control). Splenic architecture is markedly disrupted, exhibiting significant edema, extensive necrosis of parenchymal cells, abundant fibrin exudation, and loss of normal structural organization. Diffuse lymphocyte infiltration and hyperplasia are observed, with atypical morphology and visible pathological mitotic figures (arrow), characteristics consistent with the pathological features of Marek’s disease. (**B**) Group N (negative control). (**C**) Group S1 (high-dose vaccine, 5000 PFU). (**D**) Group S2 (low-dose vaccine, 500 PFU). In groups (**B**–**D**), the splenic tissues maintain clear demarcation between the red pulp and white pulp. The parenchymal cells are densely packed and arranged in an orderly manner, with no significant pathological abnormalities observed. Scale bar = 25 μm.

**Figure 5:**
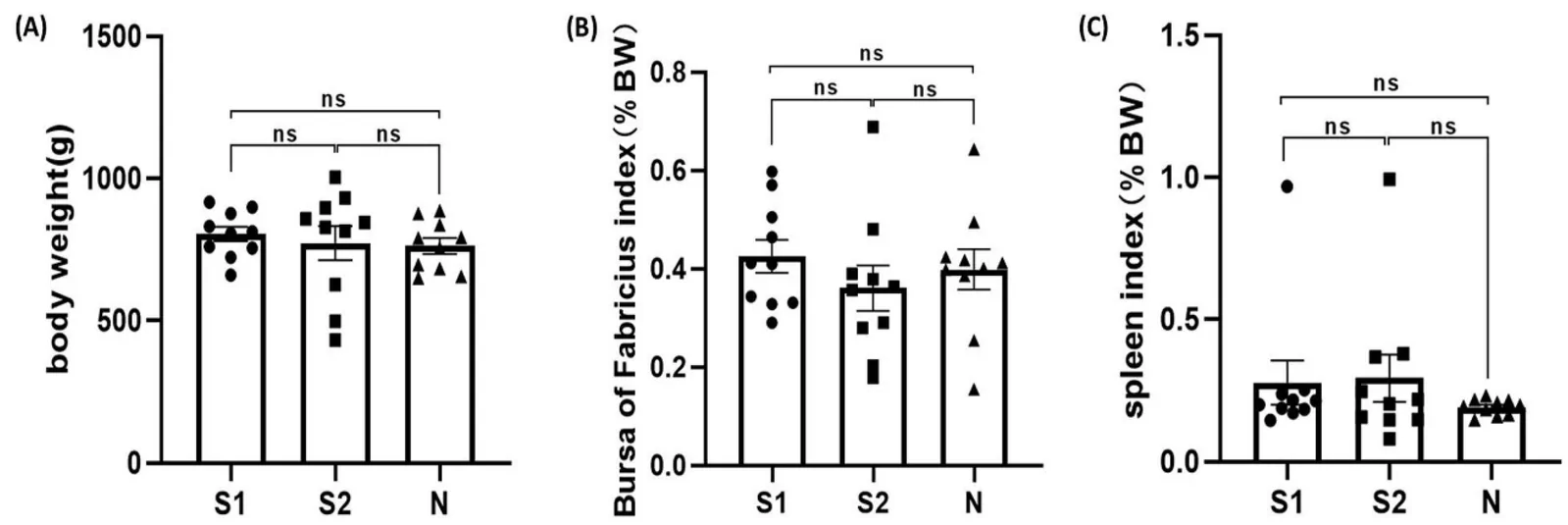
Analysis of body weight and immune organ indices at 70 days of age following Md5 challenge. Body weight (BW), bursa of Fabricius index and spleen index were determined for the high-dose vaccinated-challenged group (S1), low-dose vaccinated-challenged group (S2), and blank control group (N). All animals in challenge control group M were euthanized before reaching the 70-day experimental endpoint and were excluded from this analysis. Panels (**A)** average body weight; (**B**) bursa of Fabricius index; (**C**) spleen index. Scatter points represent data from individual chickens, and group identities are labelled on the x-axis. ns, not significant.

**Table 1:** SYBR Green qPCR primer sequences.

Primers	Sequences (5′-3′)	Product Length
gB-F	TCGACGCTTGTCCAAATCCA	103 bp
gB-R	TGGTCTATCCCCACCAGAGG	

**Table 2:** Summary of SC9-1 virus detection and quantification.

Tissue	Group	Days Post-Vaccination (dpv)					
		1 Day-of-Age (Vaccination)	3 dpv	5 dpv	7 dpv	11 dpv	14 dpv
Spleen	Q1	— ^a^	3/3 ^b^	3/3	3/3	3/3	3/3
		—	1.1 × 10^4 c^	1.5 × 10^6^	2.3 × 10^5^	6.4 × 10^3^	6.7 × 10^3^
	Q2	—	3/3	3/3	3/3	3/3	3/3
		—	4.6 × 10^3^	3.7 × 10^5^	2.0 × 10^4^	6.3 × 10^3^	7.7 × 10^3^
Feather	Q1	—	0/3	3/3	3/3	—	3/3
		—	ND ^d^	7.1 × 10^2^	6.9 × 10^3^	—	3.9 × 10^2^
	Q2	—	0/3	3/3	3/3	—	3/3
		—	ND	1.5 × 10^2^	8.6 × 10^3^	—	1.5 × 10^3^

^a^. Unsampled. ^b^. Values represent the number of qPCR-positive chickens/total number of sampled chickens at each time point. ^c^. Real-time qPCR results are given as mean virus genome copies per 10^5^ cells. ^d^. ND, not detected.

## Data Availability

The original contributions presented in this study are included in the article. Further inquiries can be directed to the corresponding author.
